# Decoding Vigilance with NIRS

**DOI:** 10.1371/journal.pone.0101729

**Published:** 2014-07-17

**Authors:** Carsten Bogler, Jan Mehnert, Jens Steinbrink, John-Dylan Haynes

**Affiliations:** 1 Bernstein Center for Computational Neuroscience Berlin and Charité – Universitätsmedizin Berlin, Berlin, Germany; 2 Max Planck Institute for Human Cognitive and Brain Sciences, Leipzig, Germany; 3 Department of Neurology, Otto-von-Guericke University Magdeburg, Magdeburg, Germany; 4 Berlin NeuroImaging Center, Charité - Universitätsmedizin Berlin, Berlin, Germany; 5 Center for Stroke Research Berlin, Charité - Universitätsmedizin Berlin, Berlin, Germany; 6 Bernstein Focus Neurotechnology Berlin, Berlin Institute of Technology, Berlin, Germany; 7 Department of Machine Learning, Institute of Technology, Berlin, Germany; The University of Queensland, Australia

## Abstract

Sustained, long-term cognitive workload is associated with variations and decrements in performance. Such fluctuations in vigilance can be a risk factor especially during dangerous attention demanding activities. Functional MRI studies have shown that attentional performance is correlated with BOLD-signals, especially in parietal and prefrontal cortical regions. An interesting question is whether these BOLD-signals could be measured in real-world scenarios, say to warn in a dangerous workplace whenever a subjects' vigilance is low. Because fMRI lacks the mobility needed for such applications, we tested whether the monitoring of vigilance might be possible using Near-Infrared Spectroscopy (NIRS). NIRS is a highly mobile technique that measures hemodynamics in the surface of the brain. We demonstrate that non-invasive NIRS signals correlate with vigilance. These signals carry enough information to decode subjects' reaction times at a single trial level.

## Introduction

The ability of organisms to maintain their focus of attention and to remain alert to stimuli over prolonged periods of time is termed “vigilance” [1]. For example, during long-lasting activities such as steering a car for hours the speed and accuracy of responses to incoming stimuli like traffic signs and other cars vary over time. Obviously, in real-world scenarios any attention failures can involve high risks. So it would be useful to have a device that measures neural correlates of attentional performance in real time and in the real world. Such a device could warn when vigilance is low or even induce events that increase attention [2]. It has long been known that the neural processes underlying attentional performance can be measured with neuroimaging techniques [3]–[5].

Several studies have demonstrated the suitability of EEG and fMRI for real-world monitoring of mental states and for brain-computer-interface (BCI) applications [6]–[16]. EEG has the potential to measure fast electrical signals from the surface of the scalp. However, for attention monitoring EEG signals might not be the first choice. EEG is very sensitive to motion artifacts [17] and the preparation is tedious, although recent developments might in future decrease preparation time drastically. Furthermore, an EEG system can always be influenced by other electronic or magnetic devices, which complicate its usability in technical environments like (car or airplane) cockpits [but see 18 for an application during simulated driving]. Finally, EEG-based BCIs are very useful when high bit-rates are necessary (for example communication devices). However, for monitoring slow fluctuations of attention and vigilance such high bit-rates might not be necessary.

A different approach would be to use functional magnetic resonance imaging (fMRI). Numerous studies have demonstrated correlates of attention in BOLD signals as measured with fMRI, ranging from sensory regions to parietal and prefrontal cortex [3], [5]. However, functional magnetic resonance imaging is stationary and thus lacks the mobility needed for natural environmental settings.

Here, we assessed the possibility of attention monitoring using Near-Infrared Spectroscopy (NIRS) as a potentially suitable technique for measuring brain activity in natural environments. Similar to fMRI, NIRS measures the blood oxygenation level of the superficial layers from the surface of the human brain and can distinguish between concentration changes of oxygenated and deoxygenated hemoglobin (HbO and HbR). Thereby, it measures an effect comparable to the blood oxygenation level dependent (BOLD) effect in fMRI [19], [20]. While concentration of HbO is expected to increase after focal activation of the cortex due to higher blood flow, HbR is washed out and decreases [21], [22]. So NIRS might potentially be able to register attention-related BOLD-signals near the surface of the brain, particularly in parietal and prefrontal cortex. Although NIRS has the substantial benefits of low-costs, easiness to handle and, especially, its mobility, it suffers from spatial limitations as compared to fMRI. While the spatial resolution can be enhanced to a sub-centimeter space [23]–[26], the depth in which hemodynamics can be measured is physically limited to the upper layer of the cortex. Thereby, fMRI remains the ‘gold standard’ in localizing hemodynamics.

NIRS has been successfully used to investigate the neural signatures of performing vigilance tasks, typically by contrasting a task with a resting or control condition [27]–[33]. It has been shown that typically the involvment of the right hemisphere is stonger relative to the left hemisphere in vigilance tasks [27], [28], [31], however the involvment of both hemispheres also depends on the vigilance task used [28], [31]. Interestingly NIRS has been used in ecologically valid environments to investigate vigilance [32]. However, the goal in these previous studies was to identify regions that show differences in brain activity between performing a vigilance task compared to some kind of baseline condition, for example an easy control task or rest. Importantly, a region that is active during the subjects' involvement in a task that requires sustained attention, does not necessarily also reflect performance fluctuations during the execution of the task. It could be possible that depending on the chosen vigilance task, a brain region shows an increased response that is related to specific properties of the task that is not necessarily related to vigilance, for example difficulty. Therefore, in the present study we are interested in measuring and relating fluctuations in the NIRS signal to performance fluctuations during the performance of a task that requires sustained attention. With this approach we can directly test, whether activity in a specific brain region reflects a potential measure for vigilance that is not confounded with differences between the vigilance and a control task (for example difficulty). Furthermore, our approach is closer to realistic scenarios in which it would be necessary to monitor performance fluctuations during vigilance tasks and not simply detect whether a monitored subject is performing a vigilance or some other kind of task. Specifically, in the present study we want to investigate whether NIRS signals are systematically modulated by vigilance fluctuations (as measured by changes in response times of a task) (Analysis I). Furthermore, we want to use the spatial-temporal pattern of the recorded NIRS-signal to decode single trial reaction times (Analysis II).

Another neuroimaging technique that could potentially be used to associate brain signals with performance is Transcranial Doppler Sonograply (TCD). TCD has already been shown to be sensitive to critical event detection during a vigilance task [34]. TCD measures global blood flow changes while NIRS is a more focal measure of cortical hemodynamics.

NIRS is also to some degree feasible for BCI [35]–[37]. NIRS is relatively motion artifact insensitive; it is non-invasive, portable and economic [38]. Although the hemodynamic response measured with NIRS is a slow response, also the attentional state fluctuates slowly. Thus, the time resolution of NIRS could be sufficiently high to acquire neural correlates of vigilance from the surface of the brain.

## Materials and Methods

### Subjects

9 healthy subjects (22–25 years old; 4 female) without any history of neurological or psychiatric disorders participated voluntarily in our experiment and received financial compensation. All of the participants had normal or corrected to normal vision and were right handed as indexed by self-report. One subject had to be excluded from the analysis because of technical problems during the measurement. The study was approved by the Ethics Committee of the Universitätsklinikum Leipzig and all participants gave their written consent prior to the experiment.

### Experiment

**Figure 1:**
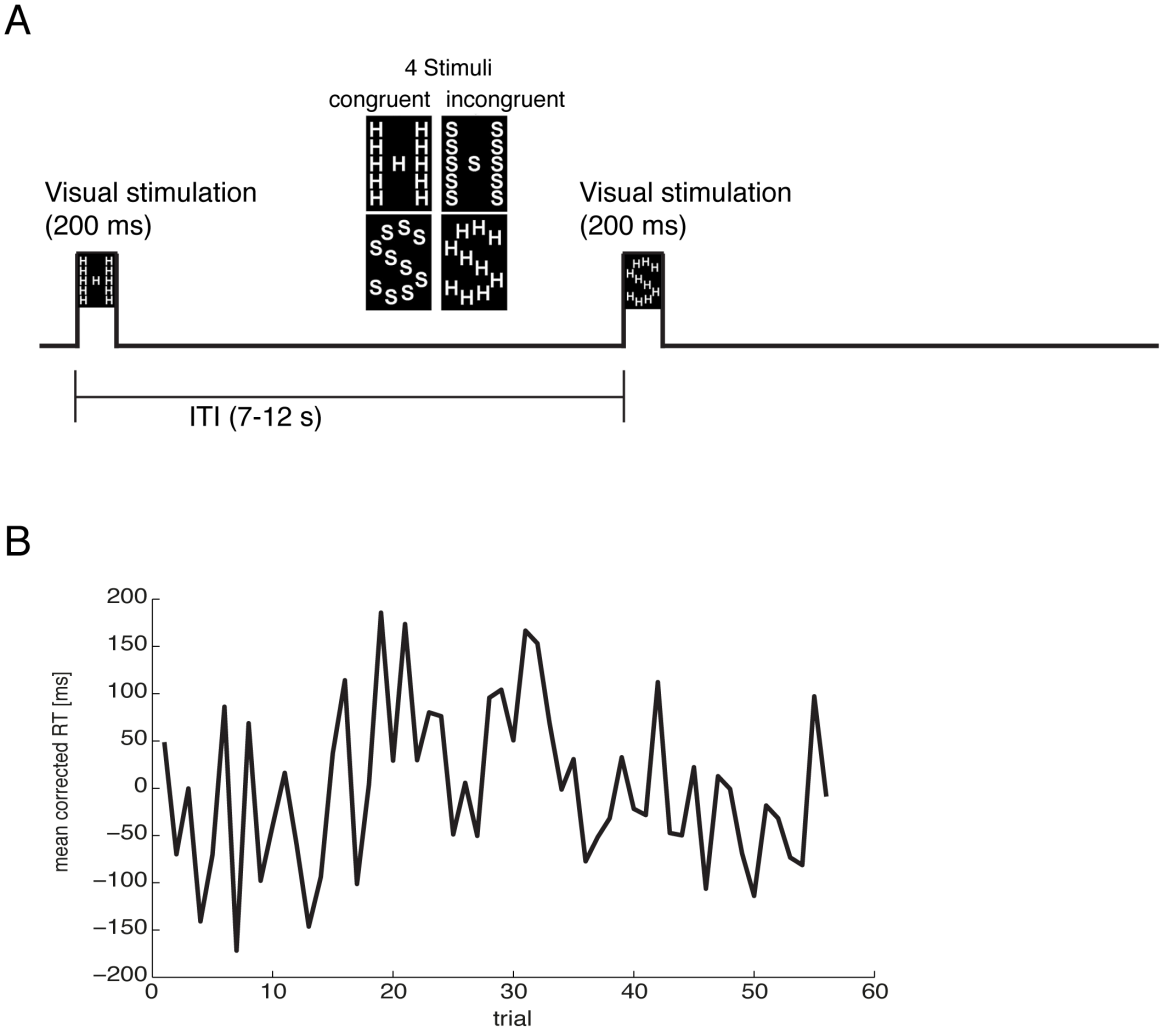
Experimental paradigm. (A) One of four possible stimuli (see middle) is presented visually for 200 ms. Subjects had to report the identity of the attended letter using a button-press. After a variable inter-trial-interval the next stimulus was presented. The stimuli were large (“global”) letters made up out of smaller letters (“local”). If the global and local levels spell different letters this creates a conflict and a decrease in reaction time. (B) Fluctuations in reaction time across the duration of the experiment were used as indicators of changes vigilance (shown here for one run of one subject).

The experimental paradigm used to measure changes in vigilance was chosen to be very similar to one that was presented in a previous fMRI study on attentional fluctuations [5]. This task was chosen because in this fMRI experiment slips of attention could then be compared to changes in the BOLD response [5]. FNIRS measures a similar signal like fMRI, however limited to the surface of the brain, therefore we expected to find similar modulations in the fNIRS signal. Subjects executed a global-local Stroop paradigm. The visual stimuli consisted of multiple small letters (either S or H) that were arranged so that the shape of either a large S or a large H was formed (see Figure 1). Half of the trials were congruent, i.e. the global and local letters were mapped to the same response, and the other half was incongruent. Before the start of each block subjects were instructed verbally to attend either to the local or the global letters. In the first 3 blocks subjects were instructed to attend to either the global or the local letters. Half of the subjects were instructed to attend to the global letters in the first 3 blocks and to the local letters in the subsequent 3 blocks. The other subjects were instructed to attend to the local letters in the first 3 blocks and to the global letters in the subsequent 3 blocks. Stimuli had a size of 66×108 pixel and were presented for 200 ms on a 15″ display with a resolution of 1024×768 pixel. After visual presentation subjects had to indicate the identity of the attended letter by button press. Inter-trial intervals (ITI) between visual presentations had a mean duration of 7.9 s (ranging from 7 to 12 s with an exponential decay and resulting in a duration of 474 s for one block). Each subject performed 6 blocks of the experiment during which 60 trials (30 congruent, 30 incongruent) were presented. Taken together the duration of the whole experiment (without short breaks between the blocks) was 47 m and 24 s. The subject's single trial performance (i.e. mean corrected reaction time) was used as an indicator for the current vigilance state for the further analyses. Please note, that the experiment was not aimed to investigate conflict processing during a Stroop task. Importantly, here we used the Stroop paradigm as an example of a task that requires relatively high levels of attention to perform it correctly. Therefore, we corrected for the behavioral effects, i.e. mainly reaction time differences between congruent and incongruent trials by subtracting the condition specific mean reaction times.

Furthermore, the Stroop task is a non-typical vigilance task that is, in the here presented used form, more similar to a psychomotor vigilance task [39]. Typical vigilance tasks involve a relatively rare event that has to be detected between very frequent events. Both the frequent and the rare event itself are very easy to detect [40], [41]. However, in a classical vigilance task the subject is asked to report only occurrences of the rare event. During such typical vigilance tasks a vigilance decrement is reported [42], i.e. increased reaction times and increased error rates over time. The Stroop task used here is useful for the acquisition of relatively high sampled measure of vigilance (reaction times) during a task that requires attention. However, because it is very different to typical vigilance tasks effects such as the vigilance decrement might not be observable.

### NIRS acquisition and preprocessing

**Figure 2:**
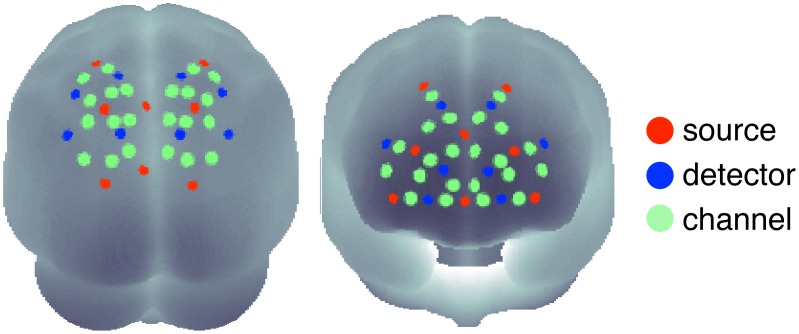
Optode positions (16 sources; red and 16 detectors; blue) and 44 measurement channels (green) on the surface of a 3D brain (left: view on parietal, right: on frontal regions). Based on previous studies [3], [5] the optode positions were focused on parietal and prefrontal cortical regions.

During the experiment, blood oxygenation at the surface of the subjects' brain was measured with a NIRS system, which consisted of 16 detectors and 16 emitters (NIRScout 16-16, NIRx Medizintechnik GmbH, Berlin, Germany) at two wavelengths (850 and 760 nm). Based on previous findings [5], we chose fiber optode positions to cover the frontal and parietal areas of the subject's head, providing a total of 44 useful channels where source and detector were at a distance of between 2 and 3 cm from each other. This arrangement is shown in Figure 2. To guarantee optimal accuracy in optode localization and convenience for the subjects, the emitters and detectors were integrated into a commercially available EEG cap (www.easycap.de) with 128 possible positions. NIRS data were continuously sampled with 6.25 Hz.

For visual inspection and demonstration, we employed the freeware MATLAB toolbox NFRI (www.jichi.ac.jp/brainlab/tools.html) by Singh and Dan [43], which takes EEG 10–20 positions, given by the EEG cap used, as references to estimate brain regions underlying the NIRS channel locations. This toolbox thereby enables statistical results for each channel to be plotted on the surface of a schematic brain as used in Figure 2. To evaluate the cortical structures underlying the NIRS channels we transformed the MNI coordinates to Talairach space [44], [45] and looked them up in a brain atlas provided by [46] (see Table 1). The channels covered brain regions that have been reported to be associated with lapses of attention [5] such as the middle frontal gyrus, precuneus, and superior parietal lobe. However, with NIRS it is only possible to measure BOLD responses on the surface of these brain regions.

**Table 1:** The MATLAB toolbox NFRI [43] was used to estimate the MNI coordinates of the used EEG 10–20 positions.

Channel #	MNI coordinate			Cortical region
	x	y	z	
ch15-16	41	62	9	Superior Frontal Gyrus
ch12-9	−30	63	20	Superior Frontal Gyrus
ch12-10	−16	64	30	Superior Frontal Gyrus
ch13-10	−5	63	35	Medial Frontal Gyrus
ch13-13	10	63	35	Superior Frontal Gyrus
ch14-13	20	64	30	Superior Frontal Gyrus
ch14-16	34	62	20	Superior Frontal Gyrus
ch9-10	−15	53	45	Superior Frontal Gyrus
ch10-13	18	52	45	Superior Frontal Gyrus
ch7-3	−32	−53	71	Superior Parietal Lobule
ch7-4	−21	−52	75	Superior Parietal Lobule
ch8-6	21	−53	75	Postcentral Gyrus
ch8-7	32	−54	72	Superior Parietal Lobule
ch4-3	−29	−65	65	Superior Parietal Lobule
ch4-4	−19	−66	70	Superior Parietal Lobule
ch5-4	−12	−65	71	Superior Parietal Lobule
ch5-6	11	−65	70	Superior Parietal Lobule
ch6-6	18	−66	70	Superior Parietal Lobule
ch6-7	28	−67	66	Superior Parietal Lobule
ch4-2	−31	−73	58	Superior Parietal Lobule
ch4-1	−18	−77	60	Precuneus
ch5-1	−11	−77	61	Precuneus
ch5-5	9	−76	60	Precuneus
ch6-5	17	−77	60	Precuneus
ch6-8	29	−74	58	Precuneus
ch1-2	−33	−85	43	Precuneus
ch1-1	−19	−88	44	Cuneus
ch2-1	−11	−85	48	Precuneus
ch2-5	8	−85	47	Cuneus
ch3-5	18	−88	45	Cuneus
ch3-8	30	−85	44	Cuneus

To evaluate the cortical structures underlying the NIRS channels the MNI coordinates were transformed to Talairach space [44], [45] and looked up in a brain atlas [46].

**Figure 3:**
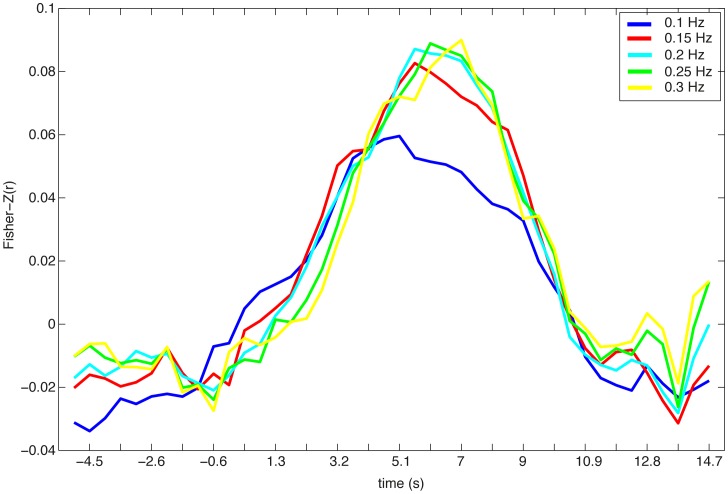
Timelines of the averaged accuracies (averaged Fisher-Z normalized correlation) of the prediction of subjects' single trial reaction times. Different low-pass cutoff frequencies were chosen during the preprocessing (0.1 Hz, 0.15 Hz, 0.2 Hz, 0.25 Hz, and 0.3 Hz). Decoding performance on low-pass filtered data with cutoff frequencies between 0.15 Hz and 0.3 Hz is very similar. When the data are low-pass filtered with a cutoff frequency of 0.1 Hz decoding performance declines.

Raw data was filtered at 0.25 Hz using a third order digital Butterworth low-pass filter. The cutoff frequency was chosen to attenuate high frequency noise and the cardiovascular signal (i.e., breathing and heartbeat). Further analysis with different cutoff frequencies (Figure 3) revealed that potential noise in the frequency of breathing (0.2–0.3 Hz) is not affecting the results of the performed decoding analysis. Attenuation changes of both measured wavelengths were transformed to concentration changes of oxy- and deoxygenated hemoglobin (HbO and HbR) using a modified Beer-Lambert law [47].

### Behavioral Data

Mean response times as well as hit rates for congruent and incongruent trials were calculated. We also calculated hit rates and response times for the six runs and for the two attention conditions (attend local or attend global) individually. For all further analysis only reaction times of correct trials were considered. In order to remove condition specific reaction time differences, we calculated the average reaction time for the congruent and for the incongruent trials for each run of each subject. Then these mean reaction times for the congruent and incongruent trials were subtracted from the individual congruent and incongruent trials respectively. Please note that after this correction it is impossible to infer from the reaction time alone, whether a trial was congruent or incongruent. However, more importantly, the corrected reaction times reflect relative performance fluctuations during the execution of the task independent of potential congruency effects.

### Analysis I: Finite Impulse Response Analysis

The preprocessed NIRS-data was analyzed using custom software. In a first analysis we implemented a general linear model (GLM) that makes no assumptions about the shape of the hemodynamic response (e.g. the shape of the hemodynamic response function). Therefore 12 consecutive stick function regressors were estimated to model the event-related hemodynamic response (ER-HR). The data were recorded with a sampling frequency of 6.25 Hz, i. e. much faster than in fMRI paradigms (typically around 0.5 Hz). Therefore, in contrast to finite impulse response (FIR) analysis for fMRI data, we modeled every 6^th^ time point with a stick function instead of impulses for each time point.

Furthermore we included 12 parametric regressors [48], one for each of the 12 onset regressors which modeled the modulation of the hemodynamic response due to variations in the mean corrected response times, resulting in a total of 24 regressors. We further refer to this set of regressors as event-related parametric modulated (ER-PM). The model used in the GLM furthermore included discrete cosine functions to attenuate slow drifts below 1/128 Hz.

The regression approach described above is similar to the analysis used in the study from Weissman [5].

### Analysis II: Decoding

The aim of Analysis II was to predict (mean corrected) single trial behavioral response times using support vector regression (SVR). For the prediction we used all 44 channels from both chromophores of the hemoglobin (HbO and HbR) resulting in 88 channels. Further preprocessing steps of the data after the application of the modified Beer-Lambert law included linear drift correction and z-normalization. Both preprocessing steps were performed separately for each run. Data used for the decoding was extracted by a sliding window that moved with a step size of 3 samples and had a width of 7 samples. The range covered by the sliding window analysis started at −5 s (−31 samples) and ended at 14.7 s (92 samples) relative to the stimulus onsets. For each trial the data from all the 88 channels x 7 time points were transformed into a vector and used as features for space-time decoding [49]. The advantage of this approach is that spatial patterns as well as temporal patterns (for example temporal gradients) can enhance the decoding performance. Correct trials from 5 of the 6 experimental runs were used to train the SVR. Trials from the run that was not used for training were used to estimate the accuracy of the prediction by calculating the correlation coefficient between predicted and actual (mean corrected) reaction times. Each experimental run was used once as test data set, i.e. we implemented a 6-fold cross validation. The correlation coefficients were Fisher-Z normalized and averaged for each subject across the cross validation steps. The accuracy (i.e. mean Fisher-Z corrected correlation) was then tested for statistical significance across subjects for each step of the sliding window.

### Analysis III: Time-frequency decoding

The same analysis as described above was also performed for individual frequencies of the NIRS data. We first calculated a wavelet transformation on the signal and separated the signal between 0.001 and 0.25 Hz into 250 frequency bands. Afterwards we performed a decoding analysis very similar to the one described above, but now for each derived frequency band separately. Instead of space-time decoding, the signal within the sliding window was averaged.

## Results

### Behavior

**Figure 4:**
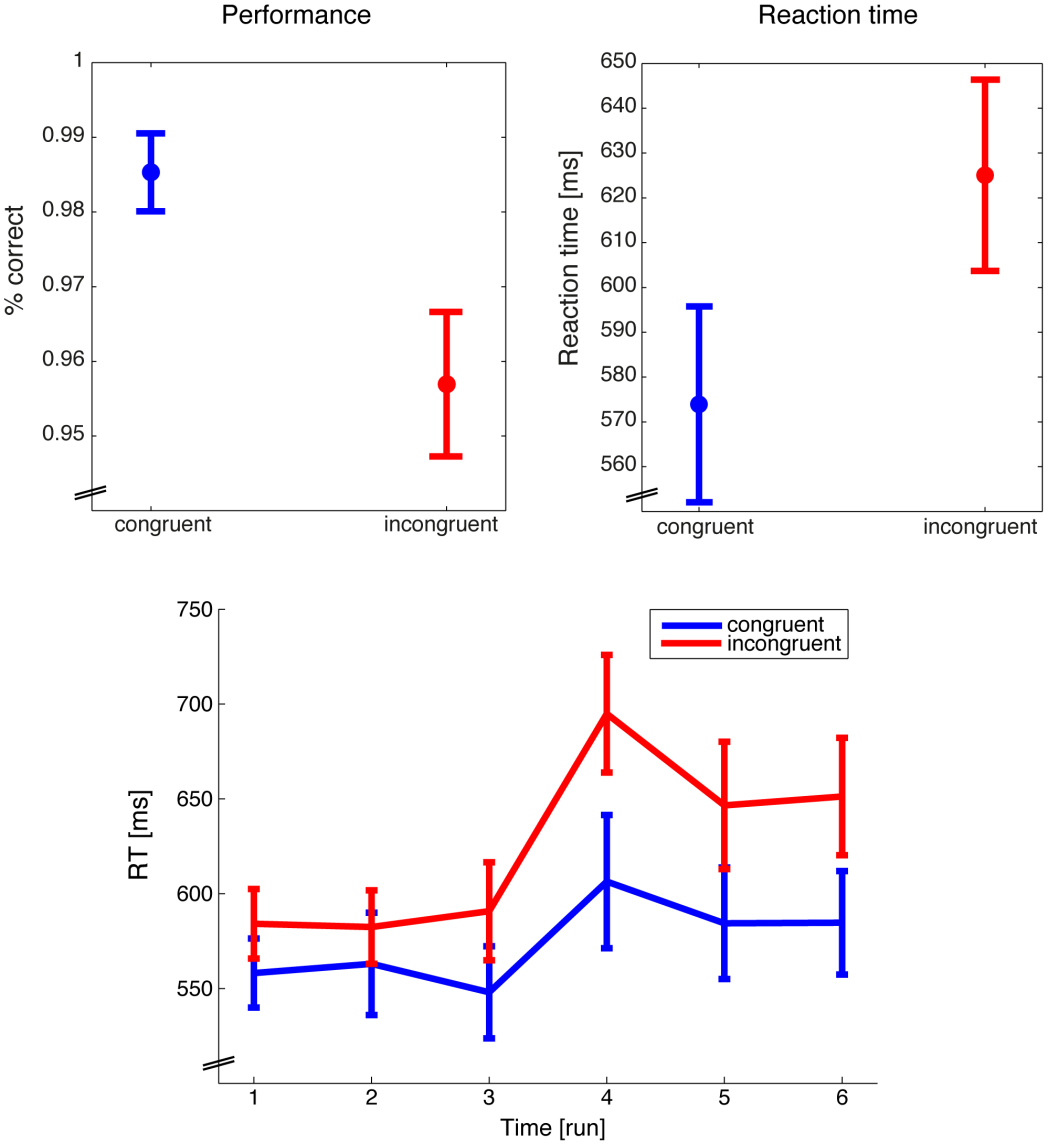
Behavioral results. Performance (left) and reaction times (right) for the congruent (blue) and incongruent (red) conditions. Responses to the congruent stimuli had a significant higher hit rate and were given faster compared to the incongruent stimuli. Reaction times to both the congruent and the incongruent stimuli were slower in the second part of the experiment (bottom). Between the third and the fourth run the instruction was to shift the attention from global to local or vice versa and potentially caused the slow response time in run 4. Error bars represent standard error of the mean.

Figure 4 shows the behavioral results. The performance (correct responses) and the response times (only for the correct responses) of the congruent trials were compared to the incongruent trials. Through the whole experiment and combined across the congruent and incongruent condition there were 10.5 errors (standard error of the mean (SEM): 2.16) ranging from a maximum of 19 to a minimum of 4 errors. In the congruent condition participants made 2.75 errors (SEM: 1.03) ranging from a maximum of 9 to a minimum of 0 errors. In the incongruent condition participants made 7.75 errors (SEM: 1.03) ranging from a maximum of 18 to a minimum of 3 errors. There were significant effects in both measures (performance: t(7) = 2.673 p<0.05, g = 1.17, response time: t(7) = −6.016 p<0.001, g = −0.79). We calculated a 2×2 repeated measurement ANOVA on the performance with the factors congruency (congruent, incongruent) and attention (global, local) that revealed a significant main effect for congruency (F(1,7) = 7.14 p<0.05) but not for attention (F(1,7) = 0.796 p = 0.402). The interaction congruency X attention was also not significant (F(1,7) = 0.092 p = 0.771). The same 2×2 repeated measurement ANOVA on the response times revealed also a significant main effect for congruency (F(1,7) = 36.198 p<0.001) but not for attention (F(1,7) = 0.737 p = 0.419) and not for the interaction (F(1,7) = 0.847 p = 0.388). Therefore, we concluded that the behavior was similar between the two attention conditions.

A 2×6 repeated measurement ANOVA with the factors congruency (congruent, incongruent) and time (run 1, 2, 3, 4, 5, and 6) was calculated on the response times to test for time sequence effects. The ANOVA again revealed a significant main effect for congruency (F(1,7) = 36.198 p<0.001) and additionally for time (F(5,35) = 3.846 p = 0.007). The interaction congruency X time was also significant (F(5,35) = 4.692 p = 0.002). Please note that after the first three runs subjects had to switch their attention from either global to local or vice versa, which potentially caused the strong response time increase in run 4.

In a final analysis we tested for sequence effects that are dependent on the global/local-attention task. Therefore, we calculated a 2×2×3 repeated measures ANOVA on the response times with the factors congruency (congruent, incongruent), attention (global, local) and time (first, second or third run with the same instruction). The ANOVA revealed a significant main effect for congruency (F(1,7) = 36.198 p = 0.001) only. All other main effects and interactions were not significant (attention F(1,7) = 0.737 p = 0.419, time F(2,14) = 1.472 p = 0.263, congruency X attention F(1,7) = 0.847 p = 0.388, congruency X time F(2,14) = 0.943 p = 0.413, attention X time F(2,14) = 0.378 p = 0.692, and congruency X attention X time F(2,14) = 2.71 p = 0.101).

Taken together we found the typical Stroop effect with lower performance and longer response times for the incongruent compared to the congruent stimuli. However, it is very important to note that we corrected the individual reaction times of each run with a condition and run specific mean, such that the mean for congruent and incongruent responses was zero. As a consequence only fluctuations around a condition specific average reaction time were used to model the NIRS response (Analysis I) and predicted by the NIRS response (Analysis II and III).

### Analysis I: Finite Impulse Response

**Figure 5:**
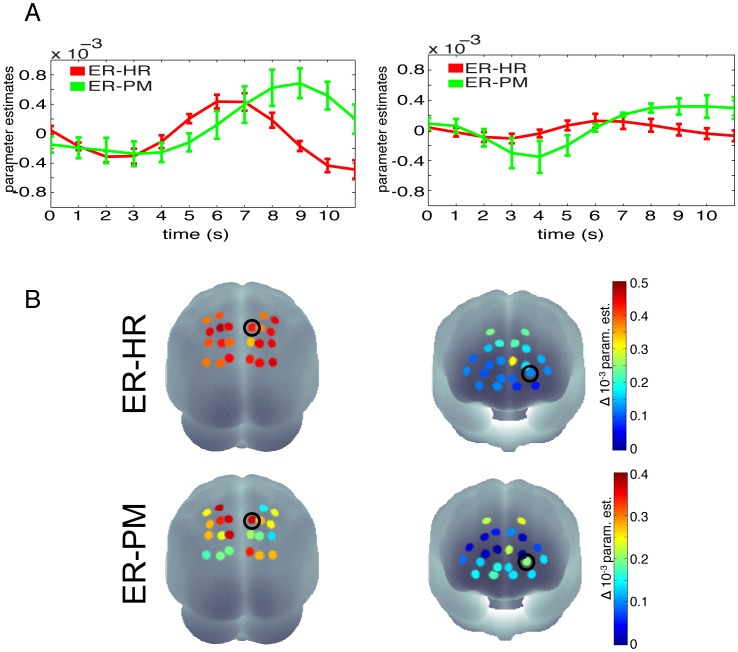
FIR-GLM parameter estimates. For illustration purposes the FIR-parameters for an HbO-channel in parietal (left) and prefrontal (right) areas are plotted. A) Time courses of FIR-parameter estimates for 2 reliable (based on a t-test on the parameter estimates) HbO-channels in parietal (left) and prefrontal (right) areas (the selected channels are marked with black circles in B below). Red: FIR parameter estimates of the ER-HR; Green: FIR parameter estimates of the ER-PM. The ER-HR can be thought of the mean averaged response to the stimuli and the ER-PM is the reaction time dependent modulation of the amplitude. For the two channels this means that for long reaction times the amplitude of early time points will be reduced and for late time points the amplitude will be increased. Please note the difference in amplitude between the prefrontal and parietal channel. B) FIR results of event-related hemodynamic response (ER-HR, top) and event-related parametric modulation (ER-PM, bottom) for the most significant time points (6 and 7 s after stimulus onset for the ER-HR and ER-PM, respectively) plotted on a 3D head surface. Black circles indicate the selected channels for which the time course is plotted in A.

The group average parameter estimates of the ER-HR revealed a classic hemodynamic response profile with a significant increase around 7 seconds after stimulus onset in most channels of oxygenated hemoglobin (HbO, Figure 5, red lines). The second set of regressors (Figure 5, green lines), for which mean corrected reaction times of the single trial were modulated by the height of the regressors' amplitude, also showed significant effects.

### Analysis II and III: Multivariate SVR

**Figure 6:**
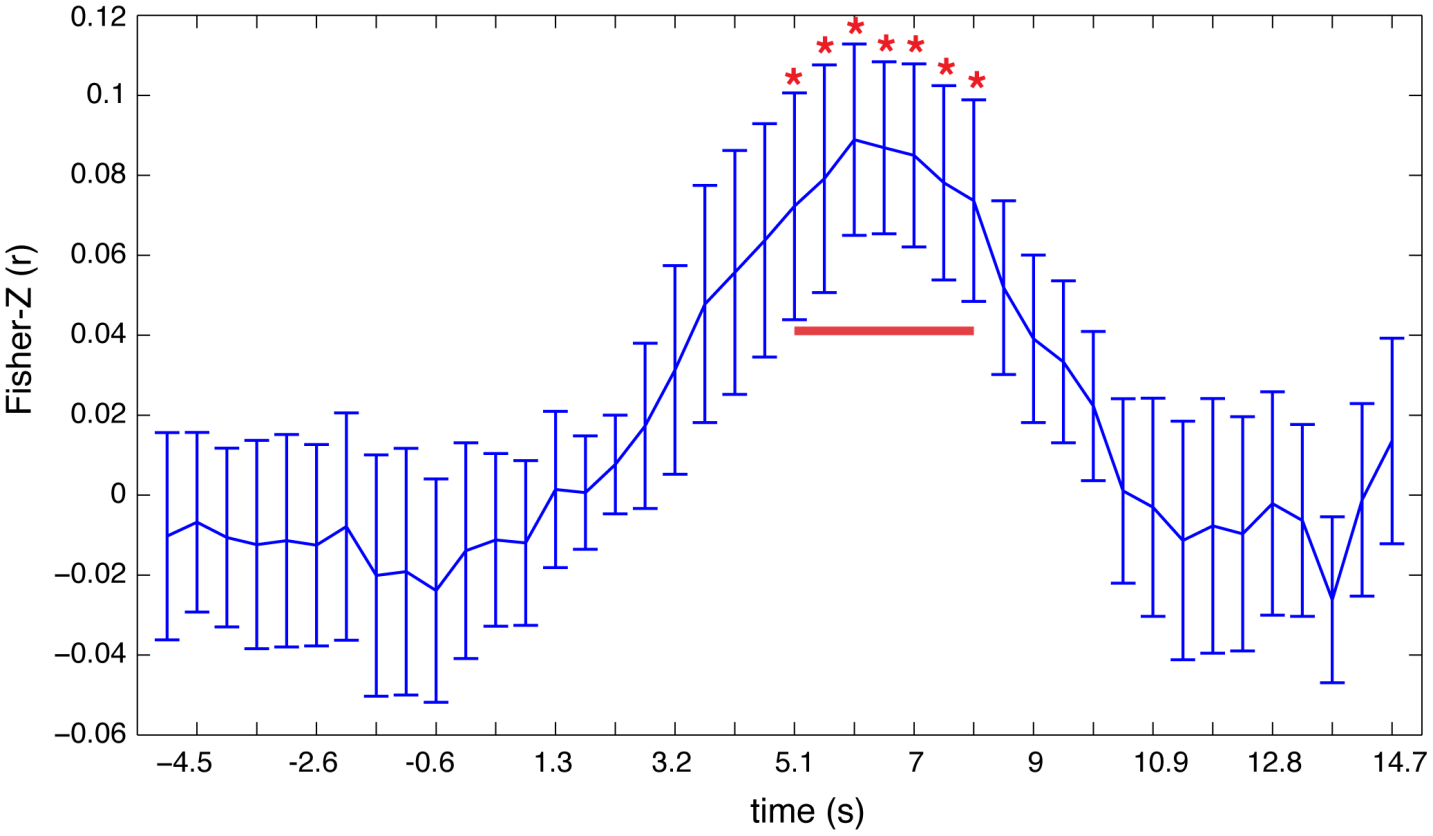
Decoding of response times. Timeline of the averaged accuracy (averaged Fisher-Z normalized correlation) of the prediction of subjects' single trial reaction times. During the time points that are marked with red asterisks (the time window that is highlighted with the red bar) decoding was significant (p<0.05; t-test on the Fisher-Z normalized correlation) above chance level.

**Figure 7:**
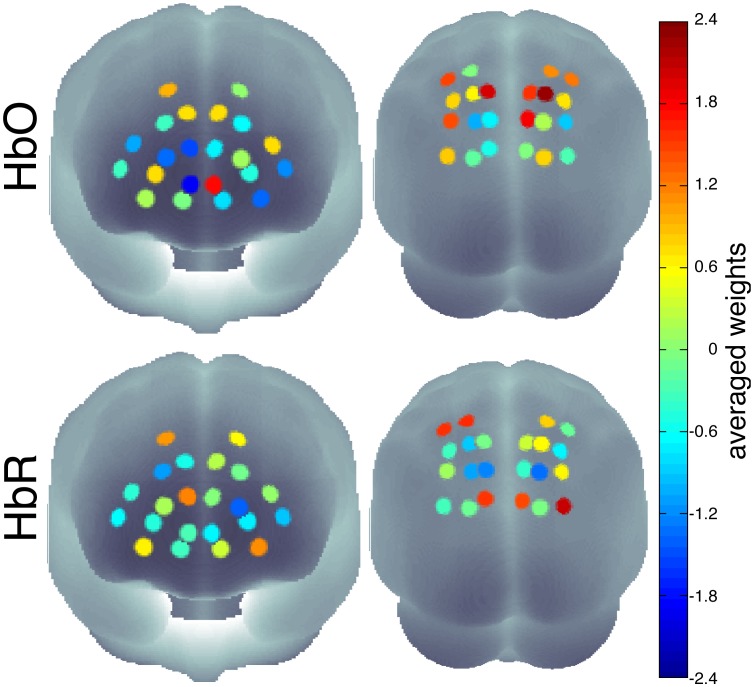
SVR weight vectors. Average SVR weight vector for the most significant time point plotted on the surface of a 3D brain. The weights constitute a filter where channels with high or low values have a strong influence on the support vector regression. We found channels contributing to the prediction in both HbR (bottom) and HbO (top), and in prefrontal (left) as well as in parietal (right) brain regions.

**Figure 8:**
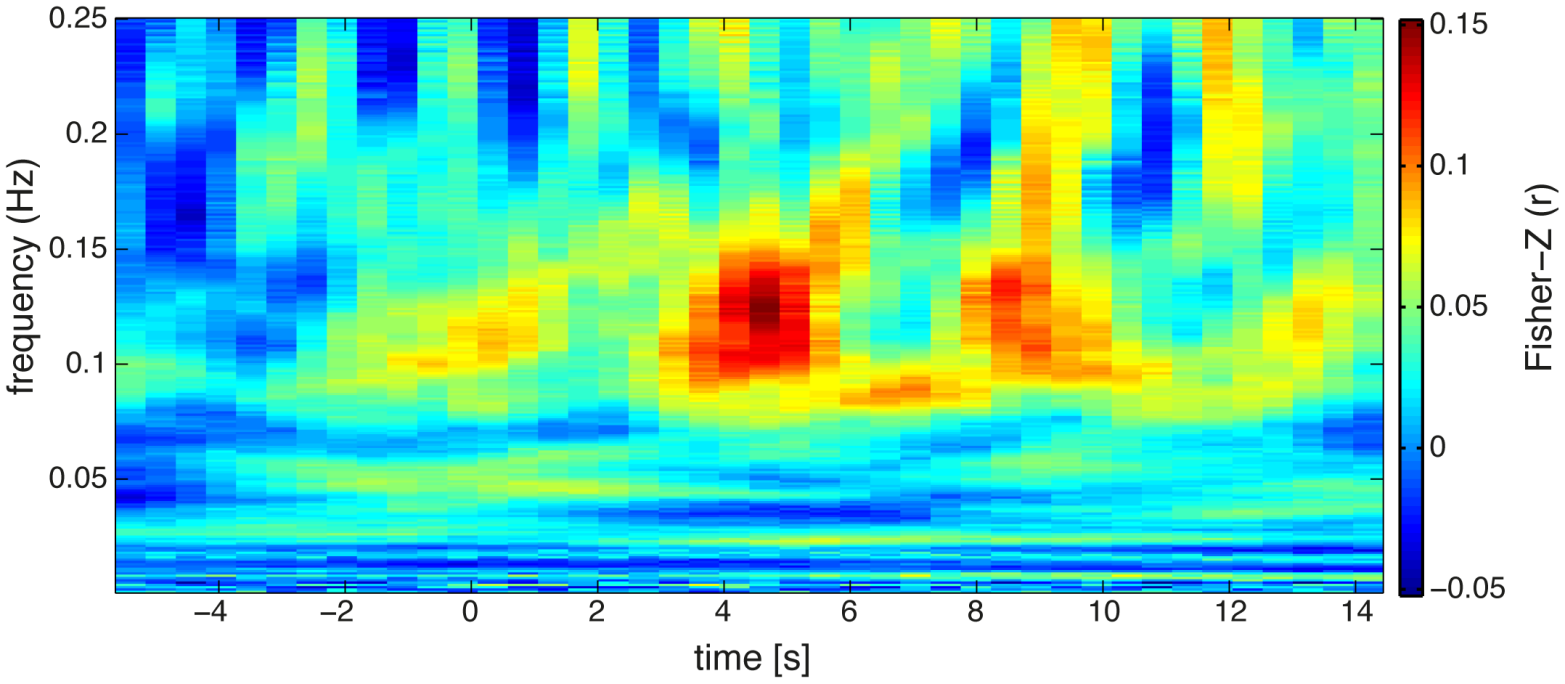
Result of the time-frequency decoding. Averaged decoding performance (averaged Fisher-Z normalized correlation) for subjects' single trial performance for individual frequencies (wavelet transformation). Most information was encoded 5 s after stimulus onset in the slow frequency range between 0.1–0.15 Hz.

Support vector regression revealed significant decoding of the reaction times between 4 and 8 seconds after stimulus onset which is around the observed time of the peak of the BOLD response as shown in Figure 6. There were no significant effects before stimulus onset which could have predicted single trial reaction times. The average inter-individual weighting vector reveals that mainly the channels from parietal areas contributed to regression (Figure 7). Please note that the neurophysiological interpretation of weight vectors of linear multivariate models can lead to wrong conclusions [50]. For example, channels that contain correlated noise can receive strong weights for removing the noise and increase the predictive power of the model. However these informative noise channels are only indirectly informative as they are used to ‘clean’ other channels. Further analysis of the spectral components (Analysis III) lead to significant effects in frequencies between 0.1 and 0.15 Hz (see Figure 8). The effect seen around this frequency range reflects the inter-trial-interval of the repetitive experimental design.

## Discussion

In the present study, we used a Stroop attention paradigm to measure fluctuations in reaction times and their neural correlates using NIRS. We were able to decode reaction time variability from NIRS signals using several approaches. Using univariate analysis techniques, we found that variations in reaction times were correlated with HbO changes in frontal and parietal regions. Results of the FIR analysis suggest that the effects are higher in HbO as compared to HbR. The average event-related hemodynamic responses (ER-HR) follow the expected canonical hemodynamic response with significant peaks between 5–8 seconds in frontal and parietal areas. The event-related parametric modulation (ER-PAR) showed that the time of peak is related to the reaction time of the subject: For slower reaction times there are also later peaks of the hemodynamic response. Neural correlates of attentional slips have been reported previously using fMRI [5]. Our results show that even at the much lower spatial resolution and with hemodynamic responses only from the surface of the brain it is possible to decode attention changes.

We showed that decoding of single trial reaction times is feasible using spatio-temporal patterns of HbR/HbO changes. Significant group statistic results were gained in the time range of 6–9 seconds after stimulus onset (Figure 6). This is also the time where the hemodynamic response is strongest and where we found strong univariate parametric modulations of the reaction times. However, we were not able to predict reaction times before stimulus onset. It has been reported previously that neural responses before stimulus onset effect responses to a stimulus [51], [52]. However, usually the pre-stimulus effect is small and only found by averaging many trials.

It might be possible that the NIRS signal reflects a recognition signal of slow (and fast) response performance. In other words it might be possible that the subject's awareness of slow/fast responses was decoded. However, we think that this is very unlikely because we decoded the single trial reaction times (condition specific mean corrected) of a whole block. It seems extremely unlikely that participants are aware of the specific deviation of their responses from the condition specific mean for each of their given responses. Furthermore, the participants might be aware when they have responded very slow but for a successful decoding of the response times this ‘awareness signal’ would have to be correlated with the actual given response times. However, a temporal separation of the response and the signal that reflects the awareness of a slow/fast response would not be possible with fNIRS and therefore we can only speculate whether the observed fNIRS signal reflects a recognition signal of slow response performance. In further studies the combination of fNIRS with EEG with much higher temporal resolution could be used to approach specifically this interesting question.

Our decoding accuracy was significant, but not very high. One of the reasons for this could be that we measured vigilance fluctuations as the variation of reaction times during a task that was performed for roughly one hour. In contrast, other studies have used tasks where subjects have to monitor very rare events, and then used the hit rate as a measure of vigilance [40]. For us such a task was not practical because we aimed for a continuous and parametric model of vigilance. However, our choice might have a disadvantage because reaction times are influenced also by other, non attention-related factors such as motivation or motor fatigue. Thus, the significant but rather low decoding performance should be considered a lower bound of the potential to decode vigilance, and future studies might want to seek neural correlates of vigilance that are robust across multiple tasks. A second reason for the low decoding performance could be that NIRS might not have sufficient information for the high accuracies required in real-world applications. In future, simultaneous other measurements for example of blood pressure, heart rate, and eye movements could be included in the decoding and potentially enhance the decoding performance.

## Summary

Taken together in the present study we show that neural correlates of vigilance can be assessed from the surface of prefrontal and parietal brain regions using NIRS. However, decoding performance on single trials is not good enough to allow for a continuous vigilance measuring device that could be of practical use yet. Therefore, further research with different tasks and simultaneous acquisition of NIRS with other physiological parameters could be fruitful.

## References

[1] Warm JS, Parasuraman R, Matthews G (2008) Vigilance requires hard mental work and is stressful. Hum Factors 50: 433–441. 18689050 10.1518/001872008X312152

[2] Nelson JT, McKinley RA, Golob EJ, Warm JS, Parasuraman R (2014) Enhancing vigilance in operators with prefrontal cortex transcranial direct current stimulation (tDCS). Neuroimage 85 Pt 3: 909–917 10.1016/j.neuroimage.2012.11.061 23235272

[3] Corbetta M, Shulman GL (2002) Control of goal-directed and stimulus-driven attention in the brain. Nat Rev Neurosci 3: 201–215 10.1038/nrn755 11994752

[4] Mangun GR, Hillyard SA (1987) The spatial allocation of visual attention as indexed by event-related brain potentials. Hum Factors 29: 195–211. 3610184 10.1177/001872088702900207

[5] Weissman DH, Roberts KC, Visscher KM, Woldorff MG (2006) The neural bases of momentary lapses in attention. Nat Neurosci 9: 971–978 10.1038/nn1727 16767087

[6] Birbaumer N, Ghanayim N, Hinterberger T, Iversen I, Kotchoubey B, et al (1999) A spelling device for the paralysed. Nature 398: 297–298. 10192330 10.1038/18581

[7] Cheng M, Gao X, Gao S, Xu D (2002) Design and implementation of a brain-computer interface with high transfer rates. Biomedical Engineering, IEEE Transactions on 49: 1181–1186. 10.1109/tbme.2002.803536 12374343

[8] Parra L, Alvino C, Tang A, Pearlmutter B, Yeung N, et al (2002) Linear spatial integration for single-trial detection in encephalography. Neuroimage 17: 223–230. 12482079 10.1006/nimg.2002.1212

[9] Buttfield A, Ferrez PW, Millan J (2006) Towards a robust BCI: error potentials and online learning. IEEE 14: 164–168. 10.1109/TNSRE.2006.875555 16792284

[10] Blankertz B, Dornhege G, Krauledat M, Müller KR, Curio G (2007) The non-invasive Berlin Brain-Computer Interface: Fast Acquisition of Effective Performance in Untrained Subjects. Neuroimage 37: 539–550. 17475513 10.1016/j.neuroimage.2007.01.051

[11] Dornhege G, Blankertz B, Curio G, Muller KR (2004) Boosting bit rates in noninvasive EEG single-trial classifications by feature combination and multiclass paradigms. IEEE 51: 993–1002. 10.1109/TBME.2004.827088 15188870

[12] Lee JH, Ryu J, Jolesz FA, Cho ZH, Yoo SS (2009) Brain-machine interface via real-time fMRI: preliminary study on thought-controlled robotic arm. Neuroscience Letters 450: 1–6. 19026717 10.1016/j.neulet.2008.11.024 PMC3209621

[13] Wolpaw JR, Birbaumer N, McFarland DJ, Pfurtscheller G, Vaughan TM (2002) Brain-computer interfaces for communication and control. Clinical Neurophysiology 113: 767–791. 12048038 10.1016/s1388-2457(02)00057-3

[14] Weiskopf N, Veit R, Erb M, Mathiak K, Grodd W, et al (2003) Physiological self-regulation of regional brain activity using real-time functional magnetic resonance imaging (fMRI): methodology and exemplary data. Neuroimage 19: 577–586. 12880789 10.1016/s1053-8119(03)00145-9

[15] Yoo SS, Fairneny T, Chen NK, Choo SE, Panych LP, et al (2004) Brain-computer interface using fMRI: spatial navigation by thoughts. Neuroreport 15: 1591–1595. 15232289 10.1097/01.wnr.0000133296.39160.fe

[16] Sorger B, Dahmen B, Reithler J, Gosseries O, Maudoux A, et al (2009) Another kind of “BOLD Response”: answering multiple-choice questions via online decoded single-trial brain signals. Progress in Brain Research 177: 275–292. 19818908 10.1016/S0079-6123(09)17719-1

[17] Luck SJ (2005) An Introduction to the Event-Related Potential Technique. Mit Press. 376 p.

[18] Haufe S, Treder MS, Gugler MF, Sagebaum M, Curio G, et al (2011) EEG potentials predict upcoming emergency brakings during simulated driving. Journal of Neural Engineering 8: 056001 10.1088/1741-2560/8/5/056001 21799241

[19] Kleinschmidt A, Obrig H, Requardt M, Merboldt KD, Dirnagl U, et al (1996) Simultaneous recording of cerebral blood oxygenation changes during human brain activation by magnetic resonance imaging and near-infrared spectroscopy. Journal of Cerebral Blood Flow and Metabolism 16: 817–826. 8784226 10.1097/00004647-199609000-00006

[20] Obrig H, Villringer A (2003) Beyond the visible–imaging the human brain with light. J Cereb Blood Flow Metab 23: 1–18. 10.1097/01.WCB.0000043472.45775.29 12500086

[21] Fox PT, Raichle ME (1986) Focal physiological uncoupling of cerebral blood flow and oxidative metabolism during somatosensory stimulation in human subjects. Proc Natl Acad Sci USA 83: 1140–1144. 3485282 10.1073/pnas.83.4.1140 PMC323027

[22] Logothetis NK, Wandell BA (2004) Interpreting the BOLD signal. Annu Rev Physiol 66: 735–769. 14977420 10.1146/annurev.physiol.66.082602.092845

[23] Koch SP, Habermehl C, Mehnert J, Schmitz CH, Holtze S, et al (2010) High-resolution optical functional mapping of the human somatosensory cortex. Front Neuroenergetics 2: 12 10.3389/fnene.2010.00012 20616883 PMC2899520

[24] Habermehl C, Holtze S, Steinbrink J, Koch SP, Obrig H, et al (2012) Somatosensory activation of two fingers can be discriminated with ultrahigh-density diffuse optical tomography. Neuroimage 59: 3201–3211 10.1016/j.neuroimage.2011.11.062 22155031 PMC3288812

[25] Gregg NM, White BR, Zeff BW, Berger AJ, Culver JP (2010) Brain specificity of diffuse optical imaging: improvements from superficial signal regression and tomography. Front Neuroenergetics 2 10.3389/fnene.2010.00014 PMC2914577 20725524

[26] Zeff BW, White BR, Dehghani H, Schlaggar BL, Culver JP (2007) Retinotopic mapping of adult human visual cortex with high-density diffuse optical tomography. Proc Natl Acad Sci USA 104: 12169–12174 10.1073/pnas.0611266104 17616584 PMC1924577

[27] De Joux N, Russell PN, Helton WS (2013) A functional near-infrared spectroscopy study of sustained attention to local and global target features. Brain Cogn 81: 370–375 10.1016/j.bandc.2012.12.003 23375118

[28] Helton WS, Warm JS, Tripp LD, Matthews G, Parasuraman R, et al (2010) Cerebral lateralization of vigilance: a function of task difficulty. Neuropsychologia 48: 1683–1688 10.1016/j.neuropsychologia.2010.02.014 20171235

[29] Helton WS, Ossowski U, Malinen S (2013) Post-disaster depression and vigilance: a functional near-infrared spectroscopy study. Exp Brain Res 226: 357–362 10.1007/s00221-013-3441-4 23435497

[30] Ossowski U, Malinen S, Helton WS (2011) The effects of emotional stimuli on target detection: indirect and direct resource costs. Conscious Cogn 20: 1649–1658 10.1016/j.concog.2011.08.015 21978909

[31] Stevenson H, Russell PN, Helton WS (2011) Search asymmetry, sustained attention, and response inhibition. Brain Cogn 77: 215–222 10.1016/j.bandc.2011.08.007 21920656

[32] Ayaz H, Shewokis PA, Bunce S, Izzetoglu K, Willems B, et al (2012) Optical brain monitoring for operator training and mental workload assessment. Neuroimage 59: 36–47 10.1016/j.neuroimage.2011.06.023 21722738

[33] Derosière G, Mandrick K, Dray G, Ward TE, Perrey S (2013) NIRS-measured prefrontal cortex activity in neuroergonomics: strengths and weaknesses. Front Hum Neurosci 7: 583 10.3389/fnhum.2013.00583 24065906 PMC3777133

[34] Shaw TH, Funke ME, Dillard M, Funke GJ, Warm JS, et al (2013) Event-related cerebral hemodynamics reveal target-specific resource allocation for both “go” and “no-go” response-based vigilance tasks. Brain Cogn 82: 265–273 10.1016/j.bandc.2013.05.003 23727665

[35] Tsubone T, Muroga T, Wada Y (2007) Application to robot control using brain function measurement by near-infrared spectroscopy. Conf Proc IEEE Eng Med Biol Soc 2007: 5342–5345. 18003214 10.1109/IEMBS.2007.4353548

[36] Abdelnour AF, Huppert T (2009) Real-time imaging of human brain function by near-infrared spectroscopy using an adaptive general linear model. Neuroimage 46: 133–143. 19457389 10.1016/j.neuroimage.2009.01.033 PMC2758631

[37] Fazli S, Mehnert J, Steinbrink J, Curio G, Villringer A, et al (2012) Enhanced performance by a hybrid NIRS-EEG brain computer interface. Neuroimage 59: 519–529. 21840399 10.1016/j.neuroimage.2011.07.084

[38] Piper SK, Krueger A, Koch SP, Mehnert J, Habermehl C, et al (2014) A wearable multi-channel fNIRS system for brain imaging in freely moving subjects. Neuroimage 85 Pt 1: 64–71 10.1016/j.neuroimage.2013.06.062 23810973 PMC3859838

[39] Dinges DF, Powell JW (1985) Microcomputer analyses of performance on a portable, simple visual RT task during sustained operations. Behavior Research Methods, Instruments, & Computers 17: 652–655 10.3758/BF03200977

[40] Davies DR, Parasuraman R (1982) The psychology of vigilance. Academic Press. 306 p.

[41] Warm JS (1984) Sustained attention in human performance. Chichester [West Sussex]; New York: Wiley.

[42] Mackworth NH (1948) The breakdown of vigilance durning prolonged visual search. Quarterly Journal of Experimental Psychology 1: 6–21 10.1080/17470214808416738

[43] Singh AK, Okamoto M, Dan H, Jurcak V, Dan I (2005) Spatial registration of multichannel multi-subject fNIRS data to MNI space without MRI. Neuroimage 27: 842–851 10.1016/j.neuroimage.2005.05.019 15979346

[44] Lancaster JL, Tordesillas-Gutiérrez D, Martinez M, Salinas F, Evans A, et al (2007) Bias between MNI and Talairach coordinates analyzed using the ICBM-152 brain template. Hum Brain Mapp 28: 1194–1205 10.1002/hbm.20345 17266101 PMC6871323

[45] Laird AR, Robinson JL, McMillan KM, Tordesillas-Gutierrez D, Moran ST, et al (2010) Comparison of the disparity between Talairach and MNI coordinates in functional neuroimaging data: Validation of the Lancaster transform. Neuroimage 51: 677–683 10.1016/j.neuroimage.2010.02.048 20197097 PMC2856713

[46] Lancaster JL, Woldorff MG, Parsons LM, Liotti M, Freitas CS, et al (2000) Automated Talairach atlas labels for functional brain mapping. Hum Brain Mapp 10: 120–131. 10912591 10.1002/1097-0193(200007)10:3<120::AID-HBM30>3.0.CO;2-8 PMC6871915

[47] Cope M, Delpy DT (1988) System for long-term measurement of cerebral blood and tissue oxygenation on newborn infants by near infra-red transillumination. Medical and Biological Engineering and Computing 26: 289–294. 2855531 10.1007/BF02447083

[48] Friston KJ, Price CJ, Buchel C, Frackowiak RSJ (1997) A Taxonomy of Study Design. In: Frackowiak RSJ, Friston KJ, Frith C, Dolan R, Mazziotta JC, editors. Human Brain Function. Academic Press USA. pp. 141–159.

[49] Mourão-Miranda J, Friston KJ, Brammer M (2007) Dynamic discrimination analysis: A spatial–temporal SVM. NeuroImage 36: 88–99 10.1016/j.neuroimage.2007.02.020 17400479

[50] Haufe S, Meinecke F, Görgen K, Dähne S, Haynes J-D, et al (2014) On the interpretation of weight vectors of linear models in multivariate neuroimaging. NeuroImage 87: 96–110 10.1016/j.neuroimage.2013.10.067 24239590

[51] Coste CP, Sadaghiani S, Friston KJ, Kleinschmidt A (2011) Ongoing brain activity fluctuations directly account for intertrial and indirectly for intersubject variability in Stroop task performance. Cereb Cortex 21: 2612–2619 10.1093/cercor/bhr050 21471558

[52] Zhang Y, Wang X, Bressler SL, Chen Y, Ding M (2008) Prestimulus cortical activity is correlated with speed of visuomotor processing. J Cogn Neurosci 20: 1915–1925 10.1162/jocn.2008.20132 18370597

