# Correction: Decoding Vigilance with NIRS

**DOI:** 10.1371/journal.pone.0123494

**Published:** 2015-04-01

**Authors:** The PLOS ONE Staff

In the Funding section, the grant number from the funder Bernstein Focus Neurotechnology Berlin is listed incorrectly. The correct grant number is: 01GQ0851.

The complete, correct funding information is as follows:

This work was supported by German Ministry for Research (BMBF 01GQ1001C), Bernstein Focus Neurotechnology Berlin (01GQ0851) and in part by NIH Grant nos. R42NS050007 and R44NS049734. The authors would like to thank the Max-Planck Institute for Human Cognitive and Brain Sciences (Leipzig, Germany) for providing the infrastructure and NIRx Medizintechnik GmbH (Berlin, Germany) for their support and technology. The funders had no role in study design, data collection and analysis, decision to publish, or preparation of the manuscript.
